# Effectiveness of laughter therapy with healthcare clowns on the mood of hospitalised adults

**DOI:** 10.15649/cuidarte.4375

**Published:** 2025-03-19

**Authors:** Yelsyn Mauricio Porras-Jiménez, Carmen Álvarez-Nieto, Karen Lizeth Romero-Granados, César Augusto Pinzón-Ordoñez, Isabel María López-Medina

**Affiliations:** 1 University of Jaén, Faculty of Health Sciences. Jaén, Spain. ympj0001@red.ujaen.es University of Jaén, Faculty of Health Sciences University of Jaén Jaén Spain ympj0001@red.ujaen.es; 2 University of Jaén, Faculty of Health Sciences. Jaén, Spain. calvarez@ujaen.es University of Jaén, Faculty of Health Sciences University of Jaén Jaén Spain calvarez@ujaen.es; 3 FOSCAL Clinic. Floridablanca, Colombia. karenromero540@gmail.com FOSCAL Clinic FOSCAL Clinic Floridablanca Colombia karenromero540@gmail.com; 4 FOSCAL Clinic. Floridablanca, Colombia. cesar.pinzono@gmail.com FOSCAL Clinic FOSCAL Clinic Floridablanca Colombia cesar.pinzono@gmail.com; 5 University of Jaén, Faculty of Health Sciences. Jaén, Spain. imlopez@ujaen.es University of Jaén, Faculty of Health Sciences University of Jaén Jaén Spain imlopez@ujaen.es

**Keywords:** Laughter Therapy, Wit and Humour as Topic, Mood, Hospitalisation, Internal Medicine, Risoterapia, Ingenio y Humor como Asunto, Estado de Ánimo, Hospitalización, Medicina Interna, Terapia do Riso, Senso de Humor e Humor como Assunto, Estado de Ânimo, Hospitalização, Medicina Interna

## Abstract

**Introduction:**

Hospitalised adults often have essential emotional needs during their hospital stay, highlighting the importance of incorporating emotional mobilisation therapies.

**Objective:**

To evaluate the effect of laughter therapy with healthcare clowns on the mood of 40 adults hospitalised in internal medicine.

**Materials and Methods:**

This quasi-experimental study used the transculturally adapted Scale for Mood Assessment (EVEA-H) to assess the intervention's effects.

**Results:**

Findings revealed significant improvements in participants' mood. There was a considerable reduction in levels of sadness/depression (p <0.00), anxiety (p <0.00) and anger/hostility (p < 0.00), while a significant increase in joy (p < 0.00) was observed after laughter therapy.

**Discussion:**

Current evidence supports laughter therapy with healthcare clowns as a tool to foster positive emotions, reduce negative psychological symptoms, and improve emotional well-being in various contexts, highlighting the importance of collaborative implementation with medical staff to strengthen well-being in healthcare settings.

**Conclusion:**

Laughter therapy can be an effective strategy to improve the emotional well-being of hospitalised adults by reducing negative moods and promoting positive emotions.

## Resumen

**Introducción:**

Los adultos hospitalizados suelen tener necesidades emocionales esenciales durante su estancia, lo que evidencia la importancia de incorporar terapias de movilización emocional.

**Objetivo:**

Evaluar el efecto de la risoterapia con payasos sociosanitarios en el estado de ánimo de 40 adultos hospitalizados en medicina interna.

**Materiales y métodos:**

Este estudio cuasiexperimental, utilizó la Escala para la Valoración del Estado de Ánimo adaptada transculturalmente (EVEA-H), para evaluar el efecto de la intervención.

**Resultados:**

Los hallazgos revelaron mejoras significativas en el estado de ánimo de los participantes. Hubo una reducción considerable en los niveles de tristeza/depresión (p <0,00), ansiedad (p <0,00) e ira/hostilidad (p <0,00), mientras que se observó un aumento significativo en la alegría (p <0,00) tras la risoterapia.

**Discusión:**

La evidencia actual apoya la risoterapia con payasos sociosanitarios como una herramienta para promover emociones positivas, reducir los síntomas psicológicos negativos y mejorar el bienestar emocional en diversos contextos. Se resalta la importancia de su implementación colaborativa con el personal médico para fortalecer el bienestar en entornos sociosanitarios.

**Conclusiones:**

Esta terapia puede ser una estrategia eficaz para mejorar el bienestar emocional de los adultos hospitalizados, reduciendo los estados de ánimo negativos y promoviendo las emociones positivas.

## Resumo

**Introdução:**

Adultos hospitalizados geralmente têm necessidades emocionais essenciais durante sua estadia, destacando a importância de incorporar terapias de mobilização emocional.

**Objetivo:**

Avaliar o efeito da terapia do riso com palhaços da saúde social no humor de 40 adultos hospitalizados em clínica médica.

**Materiais e métodos:**

Este estudo quase experimental utilizou a Transculturally Adapted Scale for Mood Assessment (EVEA-H) para avaliar o efeito da intervenção.

**Resultados:**

Os resultados revelaram melhorias significativas no humor dos participantes. Houve uma redução considerável nos níveis de tristeza/depressão (p < 0,00), ansiedade (p < 0,00) e raiva/hostilidade (p < 0,00), enquanto um aumento significativo na alegria (p <0,00) foi observado após a terapia do riso.

**Discussão:**

Evidências atuais apoiam a terapia do riso com palhaços de saúde social como uma ferramenta para promover emoções positivas, reduzir sintomas psicológicos negativos e melhorar o bem-estar emocional em vários contextos. Destaca-se a importância da sua implementação colaborativa com a equipe médica para fortalecer o bem-estar em ambientes de assistência social e de saúde.

**Conclusões:**

Esta terapia pode ser uma estratégia eficaz para melhorar o bem-estar emocional de adultos hospitalizados, reduzindo os estados de ânimo negativos e promovendo emoções positivas.

## Introduction

Hospitalisation is an event that not only involves the physiological care of a human being but also crucially addresses the pre-existing emotional needs and those arising during hospitalisation^1^. This event underscores the importance of integrating physical and emotional care. Various therapies focus on addressing emotions while providing clinical treatment, aiming to promote positive emotions and, at the same time, addressing negative ones to improve mood, facilitate adaptation to the hospital environment, and support disease management^2^-^4^. In this scenario, laughter therapy, delivered through healthcare clowns, is a promising intervention at the forefront of other therapies. 

Currently, research on the effect of laughter therapy provided by healthcare clowns is increasing, especially in the paediatric populations and, to a lesser extent, in adults^5^-^7^. Healthcare clowns began their professional practice in 1986, following the creation of the Big Apple Circus Clown Care^8^, and have since contributed to the recovery of children, adults and older adults through entertainment techniques^9^. From the beginning, performing in pairs has been considered important in healthcare clowning as it provides support during performances and helps prevent patients from feeling pressured to participate. This approach ensures that the therapy's effects are not conditioned by potential discomfort that may arise if a patient prefers to remain a spectator^10^. 

Healthcare clowns have traditionally been considered a children's thing. However, recent evidence presents important results in adults, particularly regarding emotions and other clinical aspects (pain management, cardiopulmonary stimulation, and endorphin release). These findings have allowed the development of promising research across various areas of knowledge, exploring their benefits from both a physical and psychological perspective^6^,^7^,^11^,^12^. Likewise, some physicians and nurses integrate the art of clowning into their clinical practice, further motivating the study of this topic. By combining art with unique aspects of each profession, they contribute to the holistic care of the human being^13^. 

Healthcare clowns play a key role in enhancing happiness by integrating recreational activities that help individuals rediscover life satisfaction^14^. Integrating this innovative practice introduces new ways to foster happiness, life satisfaction, and other positive emotions, which are vital for patients. As an available tool for coping with difficult life situations that may trigger anxiety and/or depression, healthcare clowning helps mediate these negative emotions^15^,^16^. Also, humour has been shown to reduce anger and hostility, with lower levels of these mood states observed after laughter-inducing interventions^17^. 

This emotional response results from the interaction of different processes, including subjective experience, cognitive appraisal, and physiological activation^18^. It triggers autonomic, endocrine, somatic, and central responses (release of dopamine and endorphins), causing activation of the sympathetic nervous system, changes in heart rate, skin conductance, and hormonal release (serotonin, noradrenaline and cortisol)^18^. Additionally, it has central implications, including changes in brain activity, particularly in the orbitofrontal and ventromedial cortex, as well as the cingulate cortex. These changes contribute to the subjective experience of emotions and influence behaviour and emotional regulation^19^. 

Emotional balance during adaptive processes, such as hospitalisation, can be disrupted, particularly in adult patients, who may experience emotional distress when perceiving this environment as a threat^20^. This feeling is linked to the separation from familiar surroundings due to inpatient care or the difficulty in coping with the illness^20^,^21^. In this context, hospitalisation represents an unwanted event that disrupts daily routines. It is unpredictable, as it is not part of a person's plans, and uncontrollable, as it involves relinquishing control of one's health to healthcare professionals^21^. 

Currently, social healthcare strives to promote well-being and foster quality of life, aiming to improve the patients' experiences beyond their hospital stay. This requires ensuring equitable and quality access to mental health resources to raise awareness of its importance at all life stages. Such efforts help prevent the onset of mental disorders and promote emotional well-being^22^. To achieve this, strategies that cultivate positive emotions and mitigate negative ones must be implemented. These include promoting self-care by encouraging people to take care of themselves physically and emotionally, strengthening coping skills through tools to navigate difficult situations effectively, fostering healthy social relationships to strengthen connections with others, and providing emotional support through safe spaces that allow emotional expression and mutual support^22^. These strategies are brought together through creative, sensitive, and humorous performances, where healthcare clowns integrate their theatrical, social, and artistic expertise. By serving as emotional mediators, they embody values such as love, compassion, and resilience, transforming the unfortunate reality of illness and hospitalisation of patients^23^-^25^. 

This study aims to explore the impact of laughter therapy delivered by healthcare clowns on the mood of hospitalised adults in an internal medicine service. It analyses how the presence of these laughter therapists can positively influence patients' emotional experiences. 

In this study, the following hypotheses were established:

**Hypothesis 1:** The intervention with healthcare clowns will reduce levels of anxiety, sadness/depression, and anger/hostility in hospitalised adults in the internal medicine service.

**Hypothesis 2:** The intervention with healthcare clowns will increase levels of happiness in hospitalised adults in the internal medicine service.

## Materials and Methods

**Design**

This pilot study employed a quasi-experimental longitudinal pre-post design with repeated measures. A single-session intervention using laughter therapy with healthcare clowns was conducted with 40 hospitalised patients in the internal medicine service of the University Hospital of Santander in Bucaramanga, Colombia. Mood states were assessed before and after intervention using the self-administered Mood Assessment Scale (EVEA-H) transculturally adapted for the Hispanic-American context. 

**Population and sample**

A convenience sampling method was employed, selecting 40 adult patients hospitalised in the internal medicine service of the University Hospital of Santander over a three-month period. This sampling method was chosen to account for the service’s occupancy capacity. The inclusion criteria were as follows: patients over 18 years of age, able to read/write, without cognitive impairments, with a diagnosis of any condition within the internal medicine area (including renal, cardiac, respiratory, and endocrine disorders, as well as infectious diseases), and having a minimum hospital stay of five days in the service. Patients under medical isolation measures were excluded from the study. To participate in the study, each patient provided a signed informed consent after understanding the study’s purpose and benefits. 

**Data collection instrument**

The Scale for Mood Assessment (EVEA), consisting of four subscales—sadness/depression, anxiety, happiness and anger/hostility—was used to measure the effectiveness of the intervention. Each subscale contains four randomly selected adjectives that assess how the patient "feels right now" on a Likert scale from 0 to 10^26^,^27^. The EVEA scale has a good internal consistency with a Cronbach's alpha of 0.92^28^. Since the original instrument was developed in Spain and the study was conducted in a Colombian cultural context, a transcultural scale adaptation was performed (Supplemental Material 1). 

The scale was administered to the patients in a physical format under the supervision of the researchers. Measurements were taken immediately before and after the intervention with healthcare clowns to minimise the impact of external factors on the observed results, such as potential improvements in patients' physical condition or the effects of attention and distraction from healthcare staff, which could have influenced their mood. As a strategy to control data loss, the completion rules were thoroughly explained to each participant, and any concerns that arose during the process were addressed, ensuring the collection of all data. 

Finally, an internal reliability analysis was conducted using Cronbach's alpha coefficient for each subscale. The results were as follows: sadness/depression (α = 0.87), anxiety (α = 0.83), anger/hostility (α = 0.86), and happiness (α = 0.86). These coefficients indicate good internal consistency across all assessed subscales. 

**Intervention**

The intervention, conducted by healthcare clowns in patients' rooms, was based on a methodological approach grounded in the performing art of clowning, using humour as a tool to manage emotions and establish meaningful social connections. Two artists, each with a unique style and personality, employed spontaneity and play to surprise and sensitively engage with patients, exploring human connection through their own authenticity. The iconic red nose, a distinctive symbol of the clown’s artistic role, was present in every intervention, which was always performed in pairs and adhered to international standards for clown therapy^29^. 

Each patient received a twenty-minute session in the morning, ensuring they were comfortably seated or lying in bed. Visits were carefully scheduled to avoid mealtimes and personal hygiene routines, preventing interference with these and other routine care activities, such as medication administration. The routine, specially designed for adults, included singing, comedic performances, games, jokes, and magic tricks, all delivered in a personalised and interactive manner. 

The therapists surprised the patients with their colourful attire, featuring vibrant costumes, eye-catching makeup, creative hairstyles, and their iconic clown noses. They also incorporated a variety of props, such as puppets, balls, guitars, books, and speakers, to entertain and create a festive atmosphere conducive to laughter. Additionally, to ease potential stress that might arise from establishing new interpersonal connections, patients were given the choice to participate as observers or active participants, according to their preferences and the progression of the session. All interventions throughout the study were conducted by the same pair of therapeutic clowns. 

**Data analysis**

Following the scoring and interpretation guidelines outlined in the Technical Data Sheet of the Scale for Mood Assessment (EVEA)^30^, each item was rated between 0 and 10 points, according to the value selected by the patient. To obtain the final scores for each mood state (sadness/depression, anxiety, happiness, and anger/hostility), the sum of the four adjectives in each subscale was calculated and then divided by 4. This process yielded four scores ranging from 0 to 10, representing the patient's pre- and post-intervention mood states. 

For each variable, the Shapiro-Wilk normality test was conducted to determine the statistical model used. Given that the Sadness/Depression (W=0.97, p=0.25), Anxiety (W=0.95, p=0.09), and Anger/Hostility (W=0.98, p=0.78) variables followed a normal distribution, the paired samples t-test was used as a parametric measure, and Cohen's D was estimated for effect size. The Happiness variable slightly differed from normal distribution (W=0.94, p=0.03). Therefore, a Wilcoxon signed-rank test was performed as a non-parametric measure, with the Hodges-Lehmann estimator used to determine effect size. Statistical analyses were performed at a significance level of p ≤.05 using the JASP statistical package (version 0.18.1). 

**Dataset**

The data collection forms were organised and stored on the lead researcher's computer, YMPJ. On a weekly basis, two researchers, KLRG and CAPO, entered the collected data into an Excel database. All data collected and the procedures used to obtain the results are available for free access and consultation in Mendeley Data^31^. 

**Ethical considerations**

This study was approved by the Ethics Committee of the University Hospital of Santander under ID number 104-OC-08432014. It was conducted in accordance with the ethical standards established in the Declaration of Helsinki^32^, the International Ethical Guidelines for Biomedical Research Involving Human Subjects established by the Council for International Organisations of Medical Sciences^33^, and the health research standards outlined in Resolution 008430 of October 4, 1993, issued by the Ministry of Health of the Republic of Colombia^34^. 

## Results

This study, conducted with 40 participants, provided relevant demographics and socioeconomic characteristics. Most participants were women, and the predominant age group was individuals between 45 and 59 years old. In terms of employment, a significant proportion were unemployed, while a considerable number worked in informal jobs, reflecting a notable economic vulnerability. Most participants came from low or very low socioeconomic backgrounds, which aligns with the typical profile of patients treated at the public hospital where the research was conducted. Cardiovascular diseases were the most prevalent condition among the participants. Furthermore, most patients had been hospitalised for 5 to 7 days, and only 4 participants reported having experienced some form of emotional disorder in their lifetime, although none were in an acute or chronic phase at the time of the study ( Table 1). 

**Table 1:** Study participant characteristics

Variable	n	%
Gender		
Male	19	47.50
Female	21	52.50
Age (Years)		
18-44	7	17.50
45- 59	28	70.00
60- 74	4	10.00
>75	1	2.50
Occupation		
Homemaker	4	10.00
Employee	2	5.00
Unemployed	18	45.00
Informal worker	16	40.00
Economic level		
Low/low	20	50.00
Low	16	40.00
Lower middle	3	7.50
Middle	1	2.50
Type of disease		
Cardiovascular diseases	16	40.00
Infectious diseases	4	10.00
Renal and genitourinary diseases	5	12.50
Metabolic and gastrointestinal diseases	3	7.50
Respiratory diseases	12	30.00
Length of hospital stay		
5 -7 days	29	72.50
8-14 days	6	15.00
15-21 days	3	7.50
> 22 days	2	5.00
History of emotional disorders		
Yes	4**	10.00
No	36	90.00

N: Sample size; %: percentage; (n): frequency; **: They were not in a chronic or acute phase at the time of the intervention.

Before the intervention, participants showed moderate levels of sadness/depression (pre mean=3.10), anxiety (pre mean=3.28), anger/hostility (pre mean=1.94), and happiness (pre mean=6.37). After the intervention, reductions in the levels of sadness/depression (post mean=1.65), anxiety (post mean=1.99), and anger/hostility (post mean=1.09) were observed, indicating an improvement in emotional well-being. Additionally, happiness increased (post mean=7.70), suggesting a positive impact on mood. These findings are supported by the decrease in standard deviations and coefficients of variation after the intervention, suggesting greater homogeneity in responses. Statistical significance was equal across all the analysed mood states (<0.00) (Table 2). Tests for statistical significance and effect size were also conducted. 

**Table 2:** Descriptive statistics of EVEA-H subscales before and after laughter therapy with healthcare clowns

Variable	Before		After		p-value
	Mean ± SD	SE/CV	Mean ± SD	SE/CV	
Sadness/Depression	3.10 ± 2.23	0.35 / 0.72	1.65 ± 1.91	0.30 / 1.16	< 0.00
Anxiety	3.28 ± 1.99	0.31 / 0.61	1.99 ± 1.85	0.29 / 0.93	< 0.00
Anger/Hostility	1.94 ± 1.82	0.29 / 0.94	1.09 ± 1.54	0.24 / 1.42	< 0.00
Happiness	6.37 ± 2.74	0.43 / 0.43	7.70 ± 1.87	0.30 / 0.24	< 0.00

EVEA-H: Scale for Mood Assessment adapted to the Hispano-American context; SD: Standard deviation; SE: Standard error; CV: Coefficient of variation, p-value: < 0.05 level of statistical significance.

The sadness/depression variable showed a significant difference between pre- and post-intervention measurements following laughter therapy with healthcare clowns in the studied group (t= 5.38, p < 0.00) and a large effect size (Cohen's D = 0.85, 95% CI=0.48- 1.21). Likewise, for anxiety, the test showed a statistically significant difference between pre- and post-intervention measures (t= 5.18, p < 0.00) with a large effect size (Cohen's D = 0.82, 95% CI=0.46- 1.17). These results indicate a clinically relevant impact of laughter therapy in controlling sadness/depression and anxiety in the studied individuals (Table 3). 

**Table 3:** Paired samples t-test for negative mood subscales of EVEA-H before and after laughter therapy with healthcare clowns

Pre/Post Measures	t	df	p-value	MD	SD	CI 95% SD		SMD	CI 95% SMD	
						Low	Upp		Low	Upp
Sadness (Depression)	5.38	39	< 0.00	1.45	0.27	0.90	2.00	0.85	0.48	1.21
Anxiety	5.18	39	< 0.00	1.29	0.25	0.78	1.79	0.82	0.46	1.17
Anger (Hostility)	3.62	39	< 0.00	0.85	0.24	0.37	1.33	0.57	0.23	0.90

EVEA-H: Scale for Mood Assessment adapted to the Hispano-American context; t: Student's t-test for paired samples; df: degrees of freedom; p-value: < 0.05 level of statistical significance; MD: Mean difference; SD: Standard deviation difference; CI: Confidence interval; Low: Lower; Upp: Upper; SMD: Cohen's d standardised mean difference.

Regarding the anger/hostility variable, the paired samples t-test reported a statistically significant difference between pre- and post- intervention measurements (t= 3.62, p < 0.00) with a moderate effect size (Cohen's D = 0.57, 95% CI=0.23- 0.90). This result indicates a clinically relevant moderate impact on anger/hostility levels in the studied group (Table 3). 

The results also indicated that laughter therapy with healthcare clowns had a statistically significant impact on happiness levels (W=125.00, p= <0.00), leading to an overall increase. Additionally, the negative Hodges-Lehmann estimator suggests that the median difference is less than zero, indicating that the pre-intervention happiness score tended to be lower than the post-intervention happiness score. The estimated magnitude of this change is around 1.25 units higher in the post-intervention medians using this laughter therapy (Table 4). 

**Table 4:** Wilcoxon signed-rank test for happiness subscale of EVEA-H before and after laughter therapy with healthcare clowns

Pre/Post Measures	W	z	p-value	Hodges-Lehmann estimator
Happiness	125.50	-3.41	< 0.00	-1.25

EVEA-H: Scale for Mood Assessment adapted to the Hispano-American context; W: Wilcoxon statistic; z: Z statistic associated with Wilcoxon contrast; p-value: < 0.05 level of statistical significance.

## Discussion

Despite great efforts to improve emotional healthcare and offer a holistic approach to human well-being in recent years, few studies have been found on the impact of healthcare clowns in the adult population across different contexts, including hospital settings. For this reason, this quasi-experimental pilot study aimed to explore the impact of laughter therapy with healthcare clowns on the mood of hospitalised adults, using the Scale for Mood Assessment adapted for Hispanic America (EVEA-H). 

The main distinction of this study lies in the selection of hospitalised adult patients in an internal medicine ward as the target population. This approach is uncommon, as most prior research in this field has predominantly focused on children, limiting the availability of recent studies for direct comparison. Nevertheless, existing research has included other populations, such as palliative care patients, women undergoing fertility treatments, and older adults^12^,^16^,^35^-^39^. These differences in population characteristics influence the expected emotional outcomes and the dynamics of the interventions. For instance, in the case of older adults, the decision to dispense with the red nose as part of the therapy has been documented, a practice informed by specific considerations for this population^16^.

Compared to previous studies, the quasi-experimental design employed in this research stands out due to its pre-post intervention approach with repeated measures. This methodology facilitated an accurate assessment of the immediate effects of laughter therapy on patients' moods. Similar methodologies have been used in other works, such as Friedler et al.^12^ in 2017, which implemented a randomised design to compare the effectiveness of the intervention with watching humorous videos. 

Studies, such as those conducted by Low et al.^16^ in 2013 and Ribeiro-dos-Santos et al.^37^ in 2021, also employed quasi-experimental designs with random sampling. Notably, Ribeiro-dos-Santos et al.^37^ conducted their study in a palliative care context, adopting a mixed-methods approach and conducting long-term follow-up. Both studies are characterised by the use of advanced statistical tools and multivariate analyses, which provided a more comprehensive understanding of the interventions' effects on variables such as anxiety, depression, and patient behaviours. 

In contrast, our study adopted a more direct and specific analysis, employing parametric and non-parametric tests to evaluate the immediate impact of therapeutic clowns on the mood of adult patients. Although both approaches aim to promote emotional well-being, the methodological differences highlight the diversity of study designs and contexts in which the effects of humour and laughter are explored across various populations. This topic has emerged as a highly relevant area of study today, as it is considered a key strategy for the humanisation of healthcare services, contributing to improved well-being and quality of life for patients both within and beyond hospital settings. 

Results recommending the implementation of laughter therapy intervention with healthcare clowns in adult care were found as a means of bringing emotional health benefits. A statistically significant decrease in sadness/depression levels was observed in the study group. In contrast, the results of Low et al.^16^ in 2013 did not show a statistically significant reduction in depression levels (p>0.05) among 398 older adults treated with this technique across 35 Australian nursing homes. However, the results of our study suggested that the relationship between participation and depression varies significantly over time in a positive manner (F=6.72, p=0.00). Similarly, the results obtained by Kontos et al.^35^ in 2016 showed no statistically significant evidence of depression reduction (t=-0.90, p=0.37) among 23 older adults with dementia who received the intervention. 

However, the benefits of laughter therapy on sadness/depression are supported by the study conducted by Auerbach et al.^36^ in 2014, which evaluated the emotional states induced by healthcare clowning interventions in 119 adults and found a significant reduction in sadness. Additionally, the study by Ribeiro-dos-Santos et al.^37^ in 2021, involving 16 palliative care patients, showed a significant reduction in depression levels after the intervention. Similarly, Quintero et al.^38^ in 2015 observed a reduction in depression among 49 older adults living in nursing homes, especially among those who had pre-existing depression at the beginning of the study. 

In this study, patients' anxiety levels showed a statistically significant reduction (t=5.18, p=<0.00), suggesting that healthcare clown interventions could be an effective tool for reducing the levels of anxiety levels in hospitalised adult patients. This finding was supported by the research conducted by Ribeiro-dos-Santos et al.^37^ in 2021 on 16 palliative care patients, which evidenced a statistically significant reduction in anxiety levels (p=0.01). Furthermore, Friedler et al.^12^ in 2017 examined 200 women after undergoing in vitro fertilisation and found that laughter therapy significantly reduces anxiety compared to an intervention using a humorous film. 

Regarding anger/hostility, this study evidences a statistically significant decrease, suggesting that laughter therapy provided by healthcare clowns benefits patients experiencing irritability. Our results are supported by Auerbach et al.^36^, who conducted a study in 2014 with 119 adults receiving therapy with healthcare clowns and observed a statistically significant reduction in anger/irritability. Likewise, Linge-Dahl et al.^39^ studied 41 palliative care patients in 2023 and found a reduction in bad mood/anger of the patients after the same therapy. In contrast, the study conducted by Kontos et al.^35^ in 2016 did not present statistically significant evidence among 23 older adults with dementia following this intervention. However, that study reported a statistically significant increase in joy levels^35^, aligning with the results of our study, in which statistically significant findings suggest that healthcare clown therapy could be an effective tool for increasing joy levels. Similarly, an increase in joy was evidenced in the study conducted by Linge-Dahl et al.^39^ in 2023 with 41 palliative care patients who received this laughter therapy intervention. 

Supporting our findings, Dionigi et al.^5^ in 2016 highlighted that healthcare clowns evoke positive emotions while reducing psychological symptoms related to emotional states and reducing negative emotions such as anxiety, depression and stress. Additionally, they recognise that both healthcare personnel and patients' relatives perceive the presence of healthcare clowns positively, suggesting that their implementation into healthcare settings provides significant benefits. 

Some factors influencing the results' statistically significance may be the context of application, sample size, and personal, cultural, and social characteristics, particularly in older adults and individuals with chronic conditions such as dementia, which require longer observation periods. It is important to note that older adults laugh fewer than eighty times per day, whereas those who smile the most do so up to a hundred times per day, while those who exhibit less joy rarely achieve it^40^,^41^. Additionally, a study found a relative deficit in cognitive humour comprehension among older adults^42^. As time passes, joy tends to decline, and people adopt a more serious demeanour, possibly influenced by societal expectations, which may limit their ability to appreciate humour^40^. However, in acute or palliative states, the results are significant. 

Studies suggest that interventions with healthcare clowns elicit positive emotions, reduce negative psychological symptoms, and improve emotional well-being. The evidence supports the implementation of laughter therapy with healthcare clowns as an effective strategy across various settings, highlighting the importance of continuing research in different populations and scenarios to expand the scientific evidence base and explore potential long-term effects. This intervention can contribute positive elements to the clinical experience; however, it is essential to be implemented carefully and in collaboration with medical personnel to ensure its effectiveness and suitability for clinical settings. Furthermore, it is necessary to strengthen the importance of emotional well-being across all healthcare scenarios, encouraging more healthcare professionals and other experts from other disciplines interested in training as healthcare clowns and thus integrating this promising technique into their professional practice. 

## Conclusions

Laughter therapy with healthcare clowns can be an effective strategy for reducing negative moods (sadness/depression, anxiety and anger/hostility) while increasing joy in hospitalised adult populations. Further research on the benefits of this intervention across different settings is recommended to increase scientific evidence, enabling its use to be recommended more frequently for adult care from a biopsychosocial perspective. 

**Limitations**

As a quasi-experimental study, the lack of randomisation limits the ability to establish causal relationships with certainty. Additionally, the duration of the intervention’s effects may be limited by the absence of follow-up over time. Therefore, future studies should conduct longitudinal measurements and interventions at multiple and subsequent time points. Furthermore, subgroup analyses could be conducted based on hospitalisation diagnosis, age groups, and length of hospital stay. Likewise, increasing the sample size is also recommended to improve the study population's representativeness and the results' generalisability. 

## Supplemental Material

**Supplemental Material 1. Escala de Valoración del Estado de Ánimo: Hispanoamérica (EVEA-H) **

A continuación, encontrarás una serie de frases que describen diferentes clases de sentimientos y estados de ánimo, y al lado unas escalas de 10 puntos. Lee cada frase y rodea con un círculo el valor de 0 a 10 que indique mejor cómo te SIENTES AHORA MISMO, en este momento. No emplees demasiado tiempo en cada frase y para cada una de ellas elige una respuesta.

## References

[1] 1. Kitson AL, Muntlin Athlin Å, Conroy T. International Learning Collaborative. Anything but basic: Nursing's challenge in meeting patients’ fundamental care needs. *J Nurs Scholarship.* 2014;46(5):331-39. 10.1111/jnu.12081 24758508

[2] 2. Moon H, Journ S, Lee S. Effect of laughter therapy on mood disturbances, pain and burnout in terminally ill cancer patients and family caregivers.* Cancer Nurs.* 2024;47(1):3-11. 10.1097/NCC.0000000000001162 36066344

[3] 3. El Bitar S. Laughing in the face of stress: A humour-based group drama therapy intervention to improve resilience for people in high-stress situations [Internet]. Canada: Concordia University; 2022 [cited 2023 Jun 10]. Available from https://spectrum.library.concordia.ca/id/eprint/991165/

[4] 4. Buiting H, Ravensbergen L, van Schaik C, Ho VK, Sonke GS. Small stuff, deep underlying emotions: An overview of the positive effect of laughing. *J Anesthesiol Pain Therapy.* 2022;3(1):5-7. https://www.anesthesioljournal.com/articles/small-stuff-deep-underlying-emotions-an-overview-of-the-positive-effect-of-laughing.pdf

[5] 5. Dionigi A, Canestrari C. Clowning in health care settings: The point of view of adults. *Eur J Psychol.* 2016;12(3):473-88. 10.5964/ejop.v12i3.1107 PMC4991052 27547261

[6] 6. Efrat-Triester D, Altman D, Friedmann E, Margalit DL, Teodorescu K. Exploring the usefulness of medical clowns in elevating satisfaction and reducing aggressive tendencies in pediatric and adult hospital wards. *BMC Health Serv Res.* 2021;21(15):1-14. 10.1186/s12913-020-05987-9 PMC7789247 33407400

[7] 7. Simoes M, Oliveira A, Pinheiro MR, Vilar M, Agante D, Pazos I, et al. Well-being biomarkers and psychological functioning of adult patients during chemotherapy treatment: The effects of hospital clowns and hosting conditions. *MedRxiv*, 2023;11. 10.1101/2023.11.22.23294770

[8] 8. Koller D, Gryski C. The life threatened child and the life enhancing clown: Towards a model of therapeutic clowning. *Evid-Based Compl Alt.* 2008;5(1):17-25. 10.1093/ecam/nem033 PMC2249744 18317544

[9] 9. Dionigi A, Ruch W, Platt T. Components and determinants of the shift between own persona and the clown persona: A hierarchical analysis. *EJHR Eur J Humour Research.* 2014;1(4):58-80. 10.7592/EJHR2013.1.4.dionigi

[10] 10. Linge L. Hospital clowns working in pairs—in synchronized communication with ailing children. *Int J Qual Stud Heal*. 2009;3(1):27-38. 10.1080/17482620701794147

[11] 11. Kurudirek F, Arıkan D. Effects of therapeutic clowning on pain and anxiety during intrathecal chemotherapy in turkey. *J Pediatr Nurs. *2020;53:e6-e13. 10.1016/j.pedn.2020.01.015 32057641

[12] 12. Friedler S, Glasser S, Levitan G, Hadar D, Sasi B, Lerner-Geva L. Patients’ evaluation of intervention by a medical clown visit or by viewing a humorous film following in vitro fertilization and embryo transfer. *Evid-Based Compl Alt.* 2017;22(1):47-53. 10.1177/2156587216629041 PMC5871203 26869229

[13] 13. Xu J, Guan YX, Liu WT, Zhang Y, Zheng J, Zhang J, et al. Intervention and application of clown care in nursing homes: A scoping review. *Aging Clin Exp Res.* 2023;35(5):937-52. 10.1007/s40520-023-02376-0 36877456

[14] 14. Zhao J, Yin H, Wang X, Zhang G, Jia Y, Shang B, et al. Effect of humour intervention programme on depression, anxiety, subjective well‐being, cognitive function and sleep quality in Chinese nursing home residents. *J Adv Nur.* 2020;76(10):2709-18. 10.1111/jan.14472 32749027

[15] 15. Sun X, Zhang J, Wang Y, Zhang X, Li S, Qu Z, et al. The impact of humor therapy on people suffering from depression or anxiety: An integrative literature review. *Brain Behav.* 2023;13(9):e3108. 10.1002/brb3.3108 PMC10498079 37340873

[16] 16. Low L, Brodaty H, Goodenough B, Spitzer P, Bell J, Fleming R, et al. The Sydney multisite intervention of LaughterBosses and ElderClowns (SMILE) study: cluster randomised trial of humour therapy in nursing homes. *BMJ Open. *2013;3(1):e002072. 10.1136/bmjopen-2012-002072 PMC3549213 23315520

[17] 17. Sakuragi S, Sugiyama Y, Takeuchi K. Effects of laughing and weeping on mood and heart rate variability. *J Physiol Anthropol Appl Human Sci.* 2002;21(3):159-65. 10.2114/jpa.21.159 12148458

[18] 18. Palmero-Cantero F. La emoción desde el modelo biológico. *REME Rev Electron Motiv Emoc.* 2003;6(13):5. http://reme.uji.es/articulos/apalmf5821004103/texto.html

[19] 19. Fernandez AM, Dufey M, Mourgues C. Expresión y reconocimiento de emociones: Un punto de encuentro entre evolución, psicofisiología y neurociencias. *Rev Chil Neuropsicol.* 2007;2(1):8-20. https://www.redalyc.org/articulo.oa?id=179317882002

[20] 20. Stein DJ, Craske MG, Rothbaum BO, Chamberlain SR, Fineberg NA, Choi KW, et al. The clinical characterization of the adult patient with an anxiety or related disorder aimed at personalization of management. *World Psychiatry.* 2021,20:336-356. 10.1002/wps.20919 PMC8429350 34505377

[21] 21. Mazorco-Salas JE, Carvajal-Hernández, PK, Villanueva-Mejía N. Experiencias de afrontamiento de la enfermedad en pacientes hospitalizados en Ibagué-Tolima. *Rev Salud Púb.* 2023,25(3):1-9. 10.15446/rsap.v25n3.107220 PMC11648377 40099292

[22] 22. World Health Organization-WHO. World mental health report: Transforming mental health for all [Internet] 2022 [cited 2023 Jul 21]. Available from https://www.who.int/publications/i/item/9789240049338

[23] 23. Bruins Slot J, Hendriks M, Batenburg R. Feeling happy and carefree: A qualitative study on the experiences of parents, medical clowns and healthcare professionals with medical clowns. *Int J Qual Stud Heal.* 2018;13(1). 10.1080/17482631.2018.1503909 PMC6116696 30156995

[24] 24. Le Roux K, Stirling-Twist J. The evolution of a clown in service: Reflections on therapeutic clowning in Canada. *Can Theatre Rev*. 2020;183:14-9. 10.3138/ctr.183.003

[25] 25. Silva MR da, Costa-Marques MC da, Penha AVX, Caires S. Constructed and disseminated behaviors of the hospital clown. *Cienc Saude Coletiva.* 2022;27(6):2449-58. 10.1590/1413-81232022276.13902021 35649031

[26] 26. Sanz J, Gutiérrez S, García-Vera MP. Propiedades psicométricas de la escala de valoración del estado de ánimo (EVEA): Una revisión. *Ans Estr.* 2014;20(1):27-49. https://www.ansiedadyestres.es/sites/default/files/rev/ucm/2014/anyes2014a3.pdf

[27] 27. Sanz J. Un instrumento para evaluar la eficacia de los procedimientos de inducción de estado de ánimo: La "escala de valoración del estado de ánimo"(EVEA). *Anal Modif Conducta.* 2001;27(111):71-110. https://dialnet.unirioja.es/descarga/articulo/7061179.pdf

[28] 28. Becerra-Canales BD, Condori-Becerra AS, Del Rio-Mendoza JR. Validez y confiabilidad de la Escala de Valoración del Estado de Ánimo, en el contexto de la pandemia por COVID-19. *Rev Cub Enf.* 2021;37:e4460. https://revenfermeria.sld.cu/index.php/enf/article/view/4460/795

[29] 29.Federación Española de Payasos de Hospital. Metodología del clown de hospital. 2023. Consulta: Noviembre 15, 2024. Disponible en: https://feph.es/metodologia/

[30] 30. Sanz J. Ficha técnica de la Escala de Valoración del Estado de Ánimo (EVEA). UCM 2013. Consulta: Octubre 26, 2022. Disponible en: https://www.ucm.es/data/cont/docs/39-2013-04-19-Ficha%20tecnica_EVEA.pdf

[31] 31. Porras-Jiménez Y-M, Álvarez-Nieto C, López-Medina I-M. Effectiveness of laughter therapy with a social-health clown on the mood of adults in the hospital context: a pilot study. *Mendeley Data*, 2024, V1. 10.17632/dg7sz3jd33.1

[32] 32. World Medical Association. Declaration of Helsinki: Ethical principles for medical research involving human subjects.* JAMA.* 2013,310(20):2191-94. 10.1001/jama.2013.281053 24141714

[33] 33. Council for International Organizations of Medical Sciences; World Health Organization. International ethical guidelines for health-related research involving humans [Internet]. 2016 [cited 2022 Jun 7];1-122. Available from https://cioms.ch/wp-content/uploads/2017/01/WEB-CIOMS-EthicalGuidelines.pdf 40523065

[34] 34. Ministerio de Salud de la República de Colombia. Resolución número 8430 de 1993: Sobre las normas científicas, técnicas y administrativas para la investigación en salud. Consulta: Noviembre 15, 2024. https://www.minsalud.gov.co/Normatividad_Nuevo/RESOLUCION%208430%20DE%201993.pdf

[35] 35. Kontos P, Miller K, Colobong R, Palma-Lazgare LI, Binns M, Low L, et al. Elder‐clowning in long‐term dementia care: Results of a pilot study. *J Am Geriatr Soc*. 2016;64(2):347-53. 10.1111/jgs.13941 PMC4846348 26889843

[36] 36. Auerbach S, Hofmann J, Platt T, Ruch WF. An investigation of the emotions elicited by hospital clowns in comparison to circus clowns and nursing staff. *EJHR Eur J Humour Research.* 2014;1(3):26-53. 10.7592/EJHR2013.1.3.auerbach

[37] 37. Santos FR dos, Pinto S, Pessalacia JDR, Luchesi BM, Silva LA da, Marinho MR. Effects of clown activities on patients eligible for palliative care in primary health care. *Rev Bras Enferm.* 2021;74(5):e20200431. 10.1590/0034-7167-2020-0431 34346954

[38] 38. Quintero Á, Henao ME, Villamil MM, León J. Cambios en la depresión y el sentimiento de soledad después de la terapia de la risa en adultos mayores internados. *Biomédica.* 2015;35(1):90-100. 10.7705/biomedica.v35i1.2316 26148038

[39] 39. Linge-Dahl L, Kreuz R, Stoffelen M, Heintz S, Ruch W, von-Hirschhausen E, et al. Humour interventions for patients in palliative care—a randomized controlled trial.* Support Care Cancer.* 2023;31(160):1-9. 10.1007/s00520-023-07606-9 PMC9925513 36781553

[40] 40. Christian R, Ramos J, Susanibar C, Balarezo G. Risoterapia: Un nuevo campo para los profesionales de la salud. *Rev Soc Peruana Med Interna.* 2004;17(2):57-64. https://revistamedicinainterna.net/index.php/spmi/article/view/238/291

[41] 41. Greengross G. Humor and Aging - A Mini-Review. *Gerontology*. 2013;59(5):448-453. 10.1159/000351005 23689078

[42] 42. Shammi P, Stuss DT. The effects of normal aging on humor appreciation. *J Int Neuropsychol Soc*. 2003;9(6):855-863. 10.1017/s135561770396005x 14632244

